# Identification of Single-Nucleotide Polymorphic Loci Associated with Biomass Yield under Water Deficit in Alfalfa (*Medicago sativa* L.) Using Genome-Wide Sequencing and Association Mapping

**DOI:** 10.3389/fpls.2017.01152

**Published:** 2017-06-29

**Authors:** Long-Xi Yu

**Affiliations:** United States Department of Agriculture-Agricultural Research Service, Plant Germplasm Introduction Testing and ResearchProsser, WA, United States

**Keywords:** linkage disequilibrium, biomass yield, drought, genotyping by sequencing, SNPs

## Abstract

Alfalfa is a worldwide grown forage crop and is important due to its high biomass production and nutritional value. However, the production of alfalfa is challenged by adverse environmental factors such as drought and other stresses. Developing drought resistance alfalfa is an important breeding target for enhancing alfalfa productivity in arid and semi-arid regions. In the present study, we used genotyping-by-sequencing and genome-wide association to identify marker loci associated with biomass yield under drought in the field in a panel of diverse germplasm of alfalfa. A total of 28 markers at 22 genetic loci were associated with yield under water deficit, whereas only four markers associated with the same trait under well-watered condition. Comparisons of marker-trait associations between water deficit and well-watered conditions showed non-similarity except one. Most of the markers were identical across harvest periods within the treatment, although different levels of significance were found among the three harvests. The loci associated with biomass yield under water deficit located throughout all chromosomes in the alfalfa genome agreed with previous reports. Our results suggest that biomass yield under drought is a complex quantitative trait with polygenic inheritance and may involve a different mechanism compared to that of non-stress. BLAST searches of the flanking sequences of the associated loci against DNA databases revealed several stress-responsive genes linked to the drought resistance loci, including leucine-rich repeat receptor-like kinase, B3 DNA-binding domain protein, translation initiation factor IF2, and phospholipase-like protein. With further investigation, those markers closely linked to drought resistance can be used for MAS to accelerate the development of new alfalfa cultivars with improved resistance to drought and other abiotic stresses.

## Introduction

Alfalfa (*Medicago sativa* L.), called “Queen of the Forages,” is a forage crop grown worldwide and is important due to its high biomass production and nutritional value. In addition to its traditional uses as an animal feed, alfalfa is also consumed by humans in the form of alfalfa sprouts and in health food products. Moreover, alfalfa is beginning to be used as a feedstock of bio-fuel production (Martin and Jung, 2010).

Changing trends toward multipurpose use is increasing demand for alfalfa. However, the production of alfalfa is challenged by adverse environmental stress factors such as drought and salinity. For instance, most alfalfa in the western United States is produced under irrigation. Water usage is high for alfalfa production in these regions. It has been documented that the water usage for California alfalfa production was higher than for other crops (Hanson et al., 2008). The costs associated with irrigation (water, pumping, maintenance, etc.) are significant, and these costs are likely to increase as populations rise in these states, increasing competition for water and power resources. In addition, climate change may adversely impact water availability. As a consequence, increased crop water use efficiency or drought tolerance will be a key factor for sustainable production of alfalfa under water-limited conditions.

Environmental factors such as drought and high salinity are frequently occurred in the arid and semi-arid regions and affect plant growth. Plants have developed several mechanisms to cope with these challenges: including adapting themselves to survive in the adverse conditions (stress-tolerance), and/or changing growth habits to avoid stress conditions (stress-avoidance). Stress-tolerant plants have evolved certain adaptive mechanisms determined by phenotypic plasticity to achieve different degrees of tolerance. Variation in stress tolerance is attributed via a series of pathways including stress perception, signal transduction, and gene expression regulation, and related metabolic pathways that contribute to tolerant plants.

Researches on the molecular bases of drought responses in alfalfa were initiated in the 1990s (Luo et al., 1991, 1992; Laberge et al., 1993). Later on, technologies such as microarrays were used for analysis of transcriptome in alfalfa response to dehydration (Chen et al., 2008). Recently, Aranjuelo et al. (2011) used proteomics and metabolite profiling and identified proteins and soluble metabolites that respond to drought in leaves and nodules of alfalfa. Most recently, RNA-seq in alfalfa varieties, Chilean and Wisfal has been performed and over 40,000 single nucleotide polymorphisms (SNPs) have been identified (Han et al., 2011; Li et al., 2012). Genome regions have been identified that were associated with forage yield in mesic environments (Li et al., 2011), flowering date and plant height (Herrmann, 2010), and drought resistance (Ray et al., 2015; Zhang et al., 2015).

Traditional and molecular breeding programs to improve drought tolerance in alfalfa have been undertaken (McKersie et al., 1996; Zhang et al., 2005; Vasconcelos et al., 2008; Suarez et al., 2009; Li et al., 2010). Modern alfalfa varieties have been used for genetic mapping (Segovia-Lerma et al., 2003; Ariss and Vandemark, 2007) and forage quality and water use efficiency (WUE; Lenssen et al., 1991; Ray et al., 1998, 2004; Segovia-Lerma et al., 2004). The method of carbon isotope discrimination was employed for measuring WUE. It has been reported that *M. sativa* ssp. falcata has higher WUE compared with other germplasm (Ray et al., 1998, 2004), although its yield was relatively low (Ray et al., 1998, 2004; Segovia-Lerma et al., 2004). Based on this finding, mapping populations were developed by crossing the *M. sativa* ssp. falcata variety Wisfal (high WUE) with Chilean germplasm (low WUE) and genetic maps has been constructed in these populations (Sledge et al., 2005). Most recently, quantitative trait loci (QTL) associated with biomass under drought stress have been identified using a traditional QTL mapping approach (Ray et al., 2015).

A large gene pool of alfalfa (>3,000 accessions) is available in the National Plant Germplasm Center. Among them, one third have been evaluated for biotic and abiotic stress resistance and other traits. However, few geneticists have tapped into this robust resource for developing cultivars. The aim of the proposed research is to identify molecular markers (genes) and germplasm for drought tolerance in the primary gene pool for developing superior alfalfa cultivars with enhanced abiotic tolerance. Traditional approaches are useful for transferring valuable QTL from unadapted germplasm into elite populations using phenotypic recurrent selection. However, this process is laborious and time-consuming. DNA marker provides a new innovative approach for rapid and inexpensive paternity testing to improve breeding gains in alfalfa. Incorporating marker-assisted selection (MAS) into plant breeding programs can accelerate progress in developing new varieties with improved resistance. Although, MAS has been widely used in the development of several important crop species, including corn, rice, and soybean, at present, it has rarely been employed for developing improved alfalfa varieties. Markers based on single nucleotide polymorphic (SNP) is the marker of choice for MAS due to their abundance, widespread distribution throughout the genome, and their potential for high-throughput detection plateform (Rafalski, 2002).

Cultivated alfalfa is an autotetraploid (2*n* = 4*x* = 32) with a genome size of 800−1,000 Mbp (Blondon et al., 1994). Alfalfa plants are highly heterozygous due to its cross pollination feature. Severe inbreeding depression precludes development of inbred line (Julier et al., 2003). It is a considerable challenge to genotype individuals with such a complicated genome. However, recent advances in next generation sequencing provide a new strategy to generate cost effective high-density genome-wide SNPs (Elshire et al., 2011). In conjunction with genome-wide association studies (GWAS) and/or genomic selection (GS; Heffner et al., 2009), more powerful platforms can be developed to improve gains in alfalfa breeding. As alfalfa cultivars are synthetic populations with broad genetic backgrounds, it would be ideal for applying them with advanced technologies such as genotyping by sequencing (GBS), association mapping (AM), and GS. Our goal is to apply genomics for developing molecular tools to accelerate breeding of alfalfa varieties with enhanced drought tolerance. We previously used GBS and GWAS and identified markers associated with drought resistance traits using a subset of the alfalfa gene pool (Zhang et al., 2015). However, it was done in controlled greenhouse conditions. As greenhouse conditions are not always the same as in the field, the field condition is more realistic since alfalfa is produced in the field in open environments. To compare gene identification in different environments, in the present study, we use the same panel of germplasm and genotyping data for identifying SNP makers associated with biomass yield under drought stress in the field.

## Materials and methods

### Plant materials

A panel of germplasm composed of 200 alfalfa accessions selected from the USDA-ARS National Plant Germplasm System (NPGS) alfalfa collection was used for evaluation and mapping loci associated with drought tolerance. A majority of germplasm were collected from Northwest regions of US and Canada including British Columbia, Saskatchewan, Manitoba, Idaho, Montana, Nebraska, Washington, and North and South Dakota. The remaining accessions were collected from different countries including Afghanistan, Algeria, Bulgaria, China, Germany, India, Lebanon, Oman, Russia, Spain, Turkey, and Yemen (Table S1).

### Field experiments

Alfalfa accessions were planted on the Roza farm at the Irrigated Agriculture Research and Extension Center of Washington State University, Prosser, WA, in 2015 and 2016. Field experiments used a randomized complete block design with three replications. Each block contains nine plants with 15 cm between rows and 5 cm between plants. They were watered regularly until the plant size was uniform (day 35–40). Subsequently, the control plants were watered regularly while drought treatment was applied to the stressed plants by withholding water during the dry season (June–September). Periodic irrigation was applied as necessary. The exact watering interval was determined during the trial through visual evaluation of wilting of the plants and measurement of soil moisture. The soil moisture was measured using ProCheck with the GS3 sensor (Decagon Devices Inc., Pullman, WA) to ensure that water deficit conditions (i.e., that the average soil water potential was below −0.03 Mpa) were maintained until harvesting.

### Phenotyping and data analysis

Early in the flowering stage, aboveground biomass was harvested, with three cuttings per year, and biomass yield was measured. A statistical analysis was conducted for each harvest. The equality of variance and means were analyzed by ANOVA. Meanwhile, a normality of distribution was tested for each harvest. Pearson correlation was calculated between harvests using trait means. All analyses were conducted using JMP13 (https://www.jmp.com/en_us/home.html).

### DNA extraction, GBS library preparation and sequencing

High molecular weight DNA was extracted from fresh young leaves of alfalfa plants using the Qiagen DNeasy 96 Plant kit (Qiagen, CA), following the manufacturer's protocol. DNA was quantified using a Nanodrop 1000 spectrophotometer (Thermo Scientific, http://www.thermoscientific.com) using the absorbance at 260 nm and quality checked using the ratio of 260/280 nm. High molecular weight DNA was subsequently used for library preparation. Two GBS libraries were prepared by digestion with the EcoT22I restriction enzyme and the digested DNA was ligated to unique nucleotide adapters (barcodes) followed by PCR amplification as described by Elshire et al. (2011). Libraries were placed in two lanes of an Illumina Hi-Seq2000 instrument at Cornell University Sequencing facility (Ithaca, NY) to be sequenced using 100-base single-end sequencing.

### Genotype calling

Sequence reads with barcodes were collapsed into a set of unique sequence tags using FASTQ. The master tags were then aligned to the reference genome of *M. truncatula* (Mt4.0 v1) according to Glaubitz et al. (2014). A tags-on-physical-map (TOPM) file containing the genomic position of each tag with a unique alignment was generated based on the physical map of the reference genome. The number of times each tag appeared in the sequencing data was scored using the barcode information. The tags by taxa (TBT) file were recorded according to the master tags listed for each sample. The information recorded in the TOPM and TBT files was then used to discover SNPs at each tag locus. The proportion of taxa covered by the tag locus, minor allele frequency, and inbreeding coefficient (FIT) were used for filtering the SNPs. After filtering, a total of 10,327 SNPs, with a mean individual depth of 27 X were obtained. The Row data of GBS were submitted to the NCBI Sequence Read Archive with bioproject ID: PRJNA287263 and biosample accession numbers: AMN03779142-SAMN03779330.

### Genome-wide association mapping and assignments of associated markers to known genes

GBS markers were further filtered with a cutoff value of 0.05 for minor allele frequency (MAF) and a missing value of 0.25. The remaining high quality SNPs were used for marker-trait association analysis with TASSEL (Bradbury et al., 2007). Marker-trait association was analyzed by a mixed linear model (MLM). A kinship (K) matrix was used for controlling population structure during association mapping. A false discovery rate (FDR) of 0.05 was used as a multiple-test correction for significantly-associated markers (Benjamini and Hochberg, 1995). To assign the associated markers to known genes, a BLAST search was carried out in the DNA database of Phytozome against the *Medicago truncatula* genome sequence, Mt4.0 v1 (http://phytozome.jgi.doe.gov/jbrowse/index.html?data=genomes%2FMtruncatula&loc) and the National Centre for Biotechnology Information (NCBI, http://www.ncbi.nlm.nih.gov/), using the flanking sequence of each significant marker as a query. Known genes linked to the significant loci were then assigned as putative candidates based on the annotation of gene functions.

## Results

### Phenotypic variations of biomass yield under water deficit and well-watered conditions

**Figure 1:**
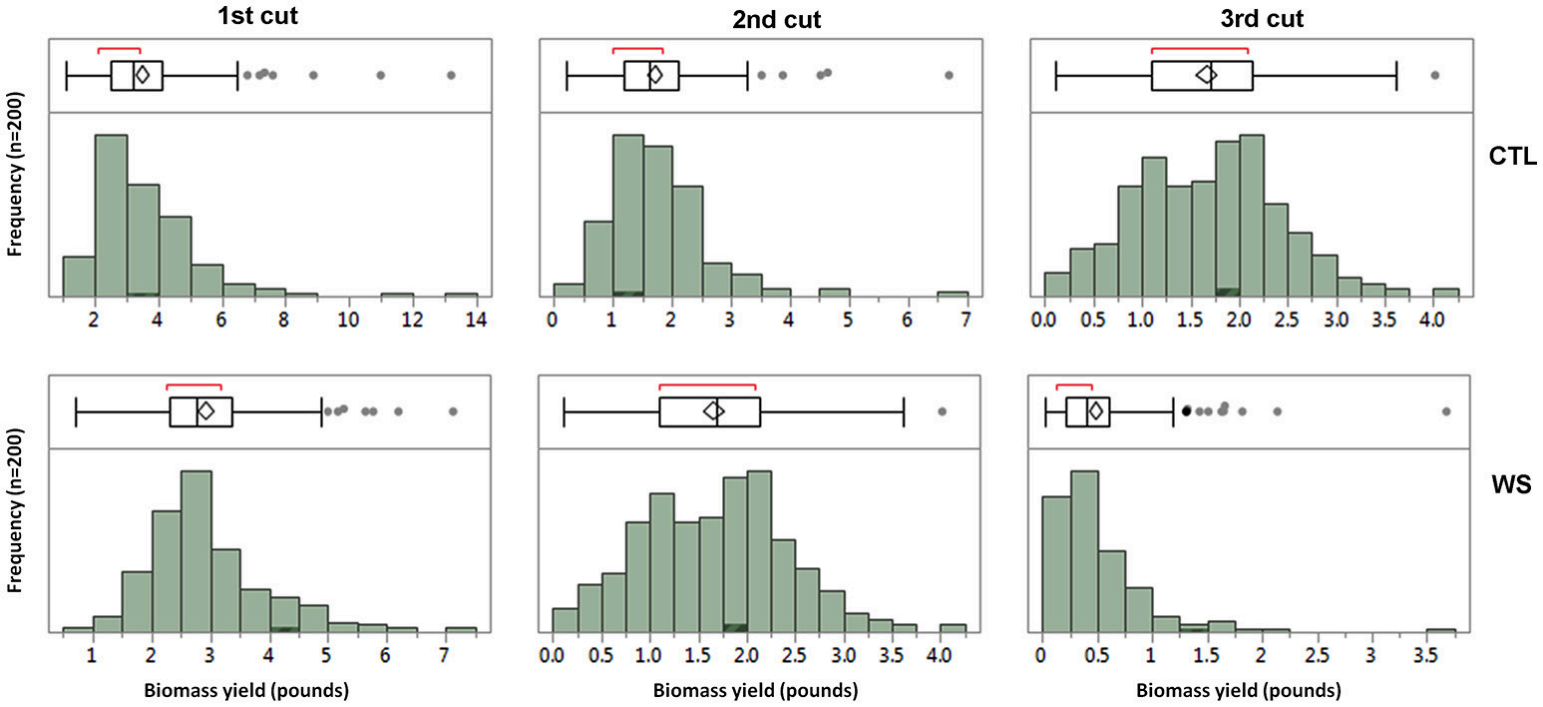
The distributions of biomass yields under well-watered control (CTL) and water stress (WS) from three cuttings in the field trial. The means of biomass in fresh weight were used for analyzing the normality of distributions in the 1st, 2nd, and 3rd cuttings. The bars of each panel show the frequency of distribution and the box plots show the pick regions of distributions.

Phenotypic data were analyzed using ANOVA for each cutting. The result is presented in Table 1. Biomass yields for all three cuttings from water deficit and control treatments were significantly different (*P* < 0.0001) among accessions. General speaking, biomass yield was significant reduced in water deficit than those of control. The parameter estimates showed variations between cuttings with highest value of 3.5 lbs in the 1st cutting of control plants and lowest of 0.48 lbs in the 3rd cutting of those of water deficit. The normality of distributions of three cuttings from control and water deficit treatments was analyzed and the result is presented in Figure 1. The mean values were higher in the 1st cuttings, with a lower mean value for the 3rd cuttings of both treatments. Correlations between cuttings were also analyzed and the result is presented in Table 2. Higher correlations (*r* = 0.47–0.82) was observed between cuttings of control plants, while lower correlations were found between treatments. However, higher correlation (*r* = 0.59) was also found between 1st cuttings of control and stressed plants (Table 2). This probably due to a small treatment effect on biomass was obtained during the 1st cuttings of the water stress treatment.

**Table 1:** Analysis of variance of biomass yields under control (CTL) and water stress (WS) in three harvests (3 cuttings).

**Parameter estimates**					**Effect test**		
**Biomass**	**Estimate (pound)**	**Std Error**	**t Ratio**	**Prob>|t|**	**Sum of Squares**	**Mean square**	**Prob > F**
1st cut CTL	3.5	0.11	31.6	<0.0001	482.19	2.44	<0.0001
2nd cut CTL	1.71	0.06	30.13	<0.0001	127.11	0.64	<0.0001
3rd cut CTL	1.67	0.05	31.88	<0.0001	107.84	0.54	<0.0001
1st cut WS	2.19	0.07	42.02	<0.0001	191.35	0.97	<0.0001
2nd cut WS	1.65	0.05	32.22	<0.0001	103.43	0.52	<0.0001
3nd cut WS	0.48	0.03	16.07	<0.0001	35.75	0.18	<0.0001

*Std, standard, Prob, probability*.

**Table 2:** Correlations of biomass yields under water stress (WS) and well-watered control (CTL) between different harvesting periods (cut).

**Correlations**	**1st cut CTL**	**2nd cut CTL**	**3rd cut CTL**	**1st cut WS**	**2nd cut WS**	**3rd cut WS**
1st Cut CTL	1	0.8197^**^	0.4738^*^	0.5881^**^	0.0423	0.0567
2nd Cut CTL	0.8197^**^	1	0.6826^**^	0.4468	0.0995	0.1472
3rd Cut CTL	0.4738^*^	0.6826^**^	1	0.3987	0.2605	0.4217
1st cut WS	0.5881^**^	0.4468	0.3987	1	0.6686^**^	0.2868
2nd cut WS	0.0423	0.0995	0.2605	0.6686^**^	1	0.4186
3rd cut WS	0.0567	0.1472	0.4217	0.2868	0.4186	1

* p < 0.01 and

** *p < 0.001, respectively*.

### Marker-trait association

**Figure 2:**
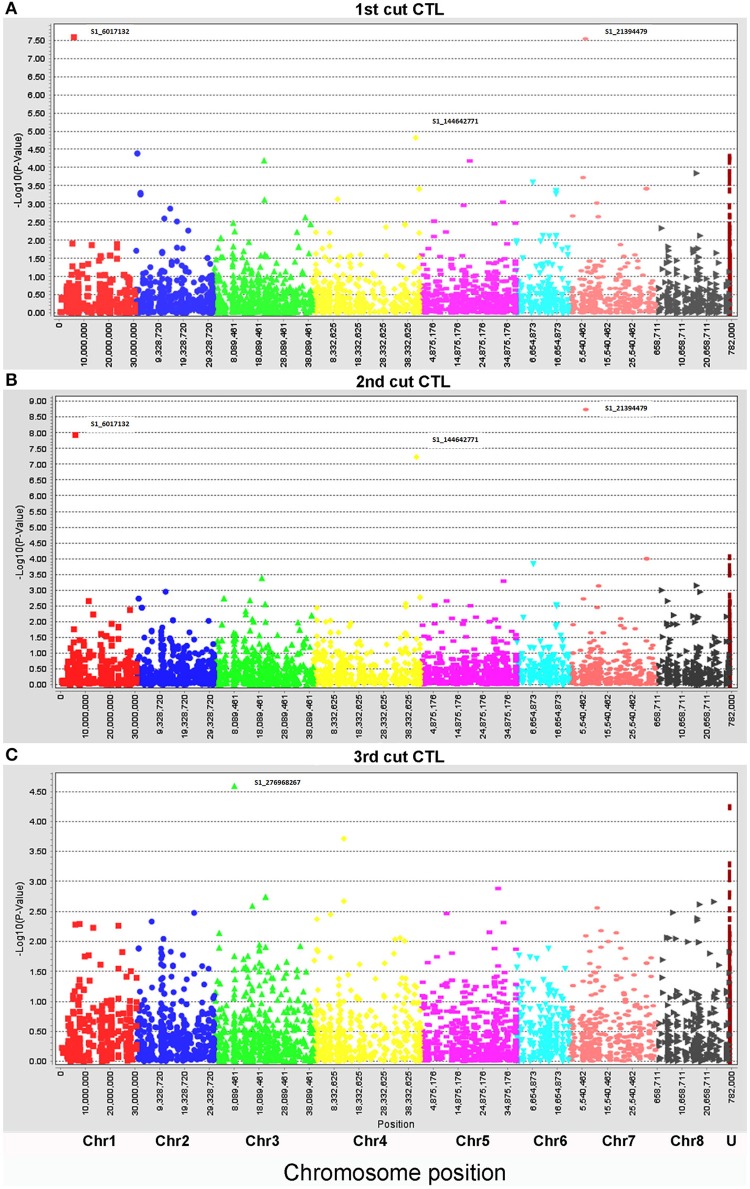
Manhattan plots of marker-trait association of biomass of alfalfa under well-watered control (CTL) in the field trial. Three harvesting data sets were used for linkage disequilibrium analyses: **(A)**, 1st harvest, **(B)**, 2nd harvest, and **(C)**, 3rd harvest. The physical positions of markers were mapped on 8 chromosomes (Chr1 to 8) and unknown positions (U) (X axes) based on the alignment of sequence tags to the reference genome (Mt 4.0 v1). The marker significances are presented as negative log *p*-values (Y axes). Significant markers at the top of each plot were indicated by marker names.

**Figure 3:**
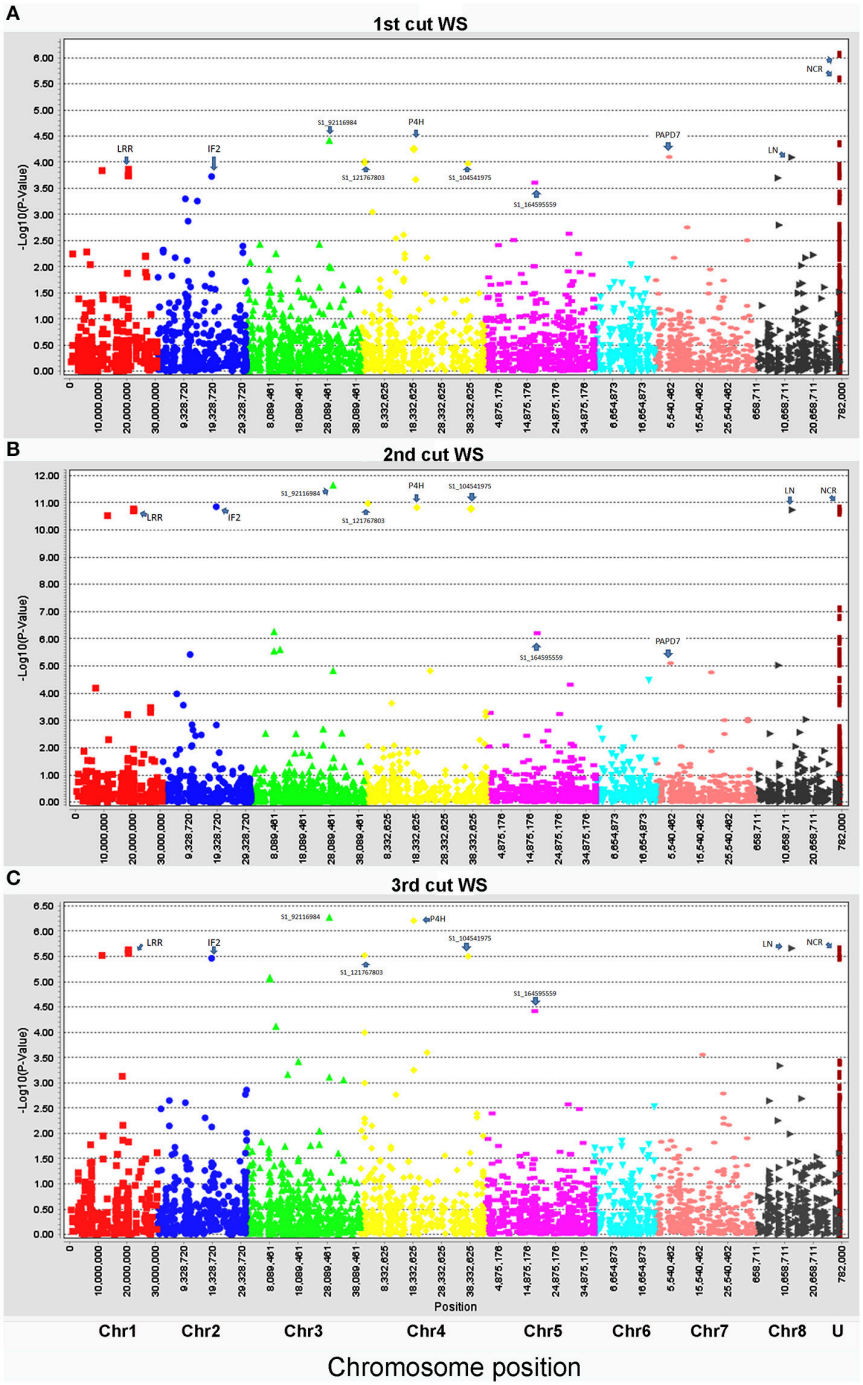
Manhattan plots of marker-trait association of biomass of alfalfa under drought stress in the field trial. Three harvesting data sets were used for linkage disequilibrium analyses: **(A)**, the 1st harvest, **(B)**, 2nd harvest, and **(C)**, 3rd harvest. The physical positions of markers were mapped on 8 chromosomes (Chr1 to 8) and unknown positions (U) (X axes) based on the alignment of sequence tags to the reference genome (Mt 4.0 v1). The marker significances are presented as negative log *p*-values (Y axes). Significant markers at the top of each plot were assigned to known genes as follows: LRR, leucine-rich repeat receptor-like kinase; IF2, translation initiation factor IF-2; P4H, prolyl 4-hydroxylase subunit alpha-like protein; PAPD7, poly(A) RNA polymerase PAPD7; LN, late nodulin; NCR, Nodule Cysteine-Rich (NCR) secreted peptide. The rest of the significant markers were labeled with their marker IDs as there were no homologs to any known genes.

Biomass yield data of three harvests were used for marker-trait association with the same genotyping data set. To compare the genotypic variation of biomass under drought and control conditions in different seasons, each harvest was analyzed independently. Under the control condition, only four markers (S1_6017132, S1_276968267, S1_21394479, and S1_144642771) were significantly associated with biomass yield (Figure 2, Table 3). Whereas, a total of 28 markers were found to be significantly associated with biomass yield under water stress (Figure 3, Table 3). Among them, 18 markers were identified in all three harvests (Table 3, highlighted with yellow) and six were identified in two harvests (Table 3, highlighted with blue). Only four markers were identified in one harvest (Table 2, unhighlighted). Since a high level of synteny was observed between the alfalfa linkage maps and the *M. truncatula* physical map (Li et al., 2014), we aligned GBS sequence tags to the reference genome of *M. truncatula* (Mt 4.0 v1). Based on the *M. truncatula* physical map, the associated markers were located at 22 loci throughout all chromosomes (Table 3, Figure 3). Since several makers were located at the same region within 60 bases of one another, we treated them as single locus, although they are listed as different markers as they have different SNP sites (Table 3). Nine markers were not mapped on the *M. truncatula* physical map. We performed BLAST searches against the DNA databases of NCBI and Phytozome using their flanking sequences as queries. Seven markers were reassigned to specific chromosomes based on the BLAST searches (Table 3, “^*^”). The remaining two markers were still unable to be aligned to the reference genome (Table 3, “U”).

**Table 3:** Comparison of SNP markers significantly associated with biomass yield under water stress (WS) and control (CTL) in different harvesting periods in the alfalfa association panel.

**Trait**	**Marker**	**SNP**	**Chr**	**1st cut**		**2nd cut**		**3rd cut**		**Candidate**
				***p*-value**	***r*^2^**	***p*-value**	***r*^2^**	***p*-value**	***r*^2^**	
Yield/WS	S1_11398797	T/G	1	1.45E-04	0.11	3.03E-11	0.31	3.03E-06	0.17	
Yield/WS	S1_20593175	A/T	1	1.82E-04	0.1	1.98E-11	0.3	2.36E-06	0.15	LRR
Yield/WS	S1_20593179	C/T	1	1.36E-04	0.1	1.70E-11	0.3	2.75E-06	0.15	LRR
Yield/WS	S1_20593180	A/G	1	1.80E-04	0.1	1.95E-11	0.3	2.73E-06	0.15	LRR
Yield/WS	S1_20593182	G/T	1	1.82E-04	0.1	1.98E-11	0.3	2.36E-06	0.15	LRR
Yield/WS	S1_368369179	T/C	1^*^	1.56E-04	0.1	1.92E-11	0.3	2.26E-06	0.15	Exp1
Yield/WS	S1_50692984	T/C	2	1.88E-04	0.1	1.43E-11	0.31	3.45E-06	0.15	IF2
Yield/WS	S1_41503160	T/A	2	5.02E-04	0.07	3.82E-06	0.12	NS	NS	
Yield/WS	S1_324999216	T/G	2^*^	4.45E-05	0.12	2.46E-11	0.31	3.32E-06	0.15	NTF
Yield/WS	S1_92116984	T/C	3	3.84E-05	0.1	2.33E-12	0.32	5.38E-07	0.15	
Yield/WS	S1_71309379	G/C	3	NS	NS	NS	NS	8.34E-06	0.14	
Yield/WS	S1_71309383	G/A	3	NS	NS	NS	NS	8.95E-06	0.14	
Yield/WS	S1_73439383	A/C	3	NS	NS	NS	NS	7.68E-05	0.12	
Yield/WS	S1_276968267	G/C	3^*^	3.92E-04	0.1	8.09E-08	0.21	3.88E-04	0.1	B3
Yield/WS	S1_276968408	G/A	3^*^	4.77E-04	0.1	8.09E-08	0.21	NS	NS	B3
Yield/WS	S1_121767803	C/A	4	5.59E-05	0.12	1.52E-11	0.31	6.23E-07	0.17	
Yield/WS	S1_104541975	G/T	4	1.00E-04	0.11	1.09E-11	0.32	3.00E-06	0.15	
Yield/WS	S1_140803955	C/T	4	1.07E-04	0.1	1.69E-11	0.3	3.14E-06	0.15	P4H
Yield/WS	S1_122448799	T/A	4	2.15E-04	0.1	NS	NS	NS	NS	PPL
Yield/WS	S1_164595559	G/A	5	2.47E-04	0.09	6.27E-07	0.17	3.83E-05	0.12	
Yield/WS	S1_326753692	T/G	6^*^	8.66E-07	0.15	6.63E-05	0.09	NS	NS	NCR
Yield/WS	S1_326753690	T/G	6^*^	2.56E-06	0.13	1.19E-04	0.08	NS	NS	NCR
Yield/WS	S1_323917811	T/C	6^*^	1.89E-04	0.1	1.98E-11	0.3	3.45E-06	0.15	
Yield/WS	S1_212244210	C/A	7	7.95E-05	0.13	7.89E-06	0.2	NS	NS	PAPD7
Yield/WS	S1_352766048	A/G	7^*^	1.16E-04	0.1	2.10E-11	0.3	3.07E-06	0.15	LTV1
Yield/WS	S1_255810503	T/C	8	8.12E-05	0.11	1.87E-11	0.31	2.18E-06	0.16	LN
Yield/WS	S1_251014656	T/C	8	2.01E-04	0.1	9.45E-06	0.13	NS	NS	NBS-LRR
Yield/WS	S1_328564258	A/G	8^*^	1.69E-04	0.1	1.61E-11	0.3	3.31E-06	0.15	KIN4
Yield/CTL	S1_6017132	A/G	1	2.63E-08	0.23	1.18E-08	0.25	NS	NS	B3
Yield/CTL	S1_276968267	G/C	3^*^	NS	NS	NS	NS	5.80E-05	0.12	B3
Yield/CTL	S1_144642771	C/T	4	1.52E-05	0.13	5.92E-08	0.19	NS	NS	XLIM
Yield/CTL	S1_213944479	G/T	7	2.89E-08	0.23	1.83E-09	0.28	NS	NS	

Chr, chromosome, “^*^”, markers that were reassigned to chromosomes based on the BLAST search. Underlined markers with the same 1st 5 digitals were located at the same locus. Markers identified in three and two harvests are highlighted in yellow and blue, respectively. The abbreviations of putative candidate genes are: LRR, leucine-rich repeat receptor-like kinase; Exp1, exportin 1-like protein; IF2, translation initiation factor IF-2; NTF, nucleotidyltransferase family protein; P4H, prolyl 4-hydroxylase subunit alpha-like protein; PPL, phospholipase-like protein; NCR, Nodule Cysteine-Rich (NCR) secreted peptide; PAPD7, poly(A) RNA polymerase PAPD7, LTV1, protein LTV1 homolog; LN, late nodulin, NBS-LRR, NBS-LRR type disease resistance protein, KIN4, serine/threonine-protein kinase KIN4; B3, B3 DNA-binding domain protein; XLI, xylose isomerase.

The markers' *p*-values ranged from E-4 to E-12 with marker S1_92116984 being the lowest (2.33E-12) and marker S1_41503160 being the highest (5.02E-4). Most significant markers were located on chromosomes 1, 3, and 4. Similar trend was found for the markers' *r*^2^ values which ranged from 0.10 to 0.32 (Table 2).

### Assigning loci associated with biomass by drought to annotated genes

To identify possible candidate genes underlying the resistance loci, The BLASTN algorithm was performed using the flanking sequences of the significant markers as queries against the Phytozome (http://phytozome.jgi.doe.gov/pz/portal.html) *M. truncatula* genome sequence (Mt4.0 v1) and the NCBI (www.ncbi.nlm.nih.gov/) nucleotide databases. Four markers (S1_20593175 to 182) at the same locus on chromosome 1 were linked to a leucine-rich repeat receptor-like kinase (LRR; Table 2, Figure 3). Another marker S1_368369179 on the same chromosome was linked to exportin 1-like protein. On chromosome 2, markers S1_50692984 and S1_324999215 were linked to a translation initiation factor (IF2) and a nucleotidyltransferase (NTF), respectively. Markers S1_140803955 and S1_122448799 on chromosome 4 were linked to prolyl 4-hydroxylase subunit and phospholipase-like protein, respectively. Two markers, S1_326753690 and S1_526753692, located at the same locus on chromosome 6 were linked to a nodule cysteine-rich (NCR) secreted peptide. On chromosome 7, markers S1_212244210 and S1_352766048 were linked to poly(A) RNA polymerase (PAPD7) and ribosome biogenesis factor (LTV1). On chromosome 8, Markers S1_255810503, S1_251014656 and 328564258 were linked to late nodulin, NBS-LRR type disease resistance protein and serine/threonine-protein kinase (KIN4), respectively. Two markers, S1_276968267 and S1_276968408 at the same locus were linked to B3 DNA-binding domain protein.

## Discussion

### Comparison of marker-trait associations for biomass yield between well-watered and water-stressed conditions

To understand genetic factors by which biomass yield is affected by water stress, phenotypic data of biomass yield under well-watered and water stress treatments were applied separately for GWAS in combination with the same genotypic data set. Different results were obtained between the two treatments. A total of 28 significant markers were associated with biomass yield in plants under water deficit, while only four markers were significantly associated with the same trait under well-watered condition. Among them, all markers identified were different expect one (S1_276968267) on chromosome 3. This marker was significant in all three harvests under water deficit and in the third harvest of well-watered condition. Different sets of markers were identified by different treatments, suggesting that different mechanisms may exist to control the yield trait under water deficit and well-watered conditions. Although the candidate gene, B3 DNA-binding domain proteins were identified in both treatments, the genetic positions of the markers linked to this gene were differently located (one on chromosome 1 and another on chromosome 3). B3 DNA-binding domain protein is a transcription factor that has been reported to play an essential role in plant growth and development under cold stress (Yamasaki et al., 2004). However, a superfamily of B3 DNA-binding domain proteins exists in plants. The ones identified in the present study by different treatments may present different members of the family with different functions.

It has been reported that a number of genes and pathways involved in the yield and yield components under abiotic stress in plants (Mickelbart et al., 2015, for review). Our result of identification of multiple loci genome-wide supports that biomass yield under water stress in alfalfa is a complex trait with polygenic inheritance. Multiple loci associated with yield trait were consistent at different harvesting periods under water deficit. For instance, among 28 markers identified within the stress treatment, 18 and 6 were identified by three and two harvests, respectively. Only 4 were identified by single harvest. It may also suggest that the experimental data and statistical procedures used in the present study are reliable.

### Comparison of drought resistance loci within the present study and between this study and the previous reports in alfalfa

Our results indicated that yield under drought is controlled by many loci over all chromosomes and this has been found in previous studies as well (Zhang et al., 2015). In the previous report, we also used GWAS and identified a group of loci associated with drought resistance index, a similar trait to biomass under drought, and the loci identified in that study were also located throughout the 8 chromosomes of alfalfa (Zhang et al., 2015). Markers identified between the two studies are not identical, however, likely due to the differences between field conditions used in the present experiment and the greenhouse conditions used previously. Whether markers identified by greenhouse or field experiments are useful for MAS or not will need further validation, which we are going to perform using different platform such haplotyping and other technologies.

The polygenic control of biomass under drought in alfalfa has been reported by different group. Recently, Ray et al. (2015) identified 25 QTLs associated with biomass under drought in two alfalfa mapping populations. They were located on all chromosomes except chromosome 7. In the present study, we identified two loci on chromosome 7. This inconsistency may due to different mapping approaches having been applied in our and Ray et al.'s studies. Ray et al. used a traditional QTL mapping approach with 600 single-dose markers, whereas we used a whole genome approach with more than 4.6 thousand single nucleotide polymorphic (SNP) markers. The SNP markers have higher coverage because of their high abundance and widespread distribution throughout the genome. Moreover, drought tolerance is a complex trait and is affected by genetic and environmental interaction. It is not surprising to have different results from different mapping populations and in different environments.

### Marker loci associated with drought resistance were linked to known stress responsive genes

Several marker loci identified in the present study were linked to genes playing roles in stress responses. Among them, the S1_20593175 locus on chromosomes 1 was linked to leucine-rich repeat receptor-like kinase. Leucine-rich repeat receptor-like protein kinases are plasma membrane proteins that are important in the transduction of various plant environmental and developmental signals (Tichtinsky et al., 2003). It has been reported that leucine-rich repeat receptor-like kinase 1 plays a role in the abscisic acid (ABA) signaling pathway in Arabidopsis (Osakabe et al., 2005). ABA is an important phytohormone that regulates plants' adaptive responses to abiotic stresses, such as drought and high salinity. It has been shown that ABA synthesis and accumulation are promoted by abiotic stress in plant cells, and ABA triggers stomatal closure in guard cells and regulates the expression of many genes with function in dehydration tolerance of plants (Finkelstein et al., 2002). Another drought responsive gene, a phospholipase-like protein, was linked to the S1_122448799 on chromosome 4. Phospholipases are involved in phospholipid-based signaling representing a component of drought-responsive signal transduction pathways (Hirayama et al., 1995; Sang et al., 2001). A serine/threonine-protein kinase was linked to S1_328564258 on chromosome 8. Plant-specific serine/threonine kinases are involved in plant response to abiotic stresses and abscisic acid signaling. It has been reported that the serine/threonine-protein kinase SOS2 is involved in the calcium-mediated regulation in plant salt tolerance in Arabidopsis (Guo et al., 2001; Zhu, 2016). Interestingly, Marker S1_251014656 on chromosome 8 was linked to a NBS-LRR type disease resistance gene. The plant NBS_LRR gene family contains a large class of disease resistance genes (DeYoung and Innes, 2006). The identification of a marker linked to the NBS-LRR genes in the present study suggests that it also involve in drought response in alfalfa. A similar finding of drought-related role for a NBS-LRR has also been reported in Arabidopsis where overexpression of the NBS–LRR gene ADR1 enhanced drought tolerance (Chini et al., 2004). It has been suggested that a signaling network exists between disease resistance and drought tolerance, and ADR1 may be involved in signal transduction in this network (Chini et al., 2004). The remaining genes had no direct function in drought tolerance, some may involve in the regulation of downstream drought tolerance genes. For instance, B3 DNA-binding domain protein linked to S1_276968267 and S1_276968408 on chromosome 3 may play a role in the transcriptional regulation for plant growth and development in response to abiotic stress (Yamasaki et al., 2004). Another example is the association of translation initiation factor IF2 with drought tolerance in the present analysis. It has been reported that phosphorylation of the α-subunit of translation initiation factor 2 is involved in the regulation of protein synthesis in response to a variety of environmental stresses (Jiménez-Díaz et al., 2013).

In conclusion, we used GBS for genome-wide genotyping and association and identified 22 marker loci associated with biomass yield under water deficit in the field in a panel of germplasm representing a diverse selection from the alfalfa gene pool. These marker loci were not presented in the well-watered control except one. The comparison of associated markers between control and stress treatments and among three sets of data obtained by three harvests showed that most of them were identical, indicating the consistency of the statistical analyses and stress treatments in the present study. The comparable results of the present study and the previous reports consistently demonstrated that loci associated with yield under drought were located throughout all 8 chromosomes, reflecting the polygenic nature of this complex trait. The BLAST search of the flanking sequences of the associated loci against DNA databases identified several stress-responsive genes linked to the drought associated loci, including serine/threonine-protein kinase, leucine-rich repeat receptor-like kinase, B3 DNA-binding domain protein, translation initiation factor IF2 and phospholipase-like protein. With further validation, those markers closely linked to drought resistance can be used for MAS to accelerate the development of new alfalfa cultivars with improved resistance to drought and other abiotic stresses.

## Author contributions

The author confirms being the sole contributor of this work and approved it for publication.

### Conflict of interest statement

The author declares that the research was conducted in the absence of any commercial or financial relationships that could be construed as a potential conflict of interest.
